# Surveillance of *Enterococcus spp*. reveals distinct species and antimicrobial resistance diversity across a One-Health continuum

**DOI:** 10.1038/s41598-020-61002-5

**Published:** 2020-03-03

**Authors:** Rahat Zaheer, Shaun R. Cook, Ruth Barbieri, Noriko Goji, Andrew Cameron, Aaron Petkau, Rodrigo Ortega Polo, Lisa Tymensen, Courtney Stamm, Jiming Song, Sherry Hannon, Tineke Jones, Deirdre Church, Calvin W. Booker, Kingsley Amoako, Gary Van Domselaar, Ron R. Read, Tim A. McAllister

**Affiliations:** 1 Lethbridge Research and Development Centre, Agriculture and Agri-Food Canada, 5403 1st Avenue South, Lethbridge, AB T1J 4P4 Canada; 2 Alberta Agriculture and Forestry, 100, 5401 1st Avenue South, Lethbridge, AB T1J 4V6 Canada; 3 Canadian Food Inspection Agency, National Center for Animal Disease, Lethbridge Laboratory, Township Rd 9-1, Lethbridge, AB T1J3Z4 Canada; 4 National Microbiology Laboratory, Public Health Agency of Canada, 1015 Arlington Street, Winnipeg, MB R3E 3R2 Canada; 5 Feedlot Health Management Services, Okotoks, AB Canada; 6 Lacombe Research and Development Centre, Agriculture and Agri-Food Canada, 6000C and E Trail, Lacombe, AB T4L 1W1 Canada; 7 Cumming School of Medicine, University of Calgary, 3280 Hospital Drive NW, Calgary, Alberta Canada; 8 Calgary Laboratory Services (CLS), Alberta Health Services, 3535 Research Rd NW, Calgary, AB T2L 2K8 Canada

**Keywords:** Microbiology, Antimicrobials, Antimicrobial resistance

## Abstract

For a One-Health investigation of antimicrobial resistance (AMR) in *Enterococcus* spp., isolates from humans and beef cattle along with abattoirs, manured fields, natural streams, and wastewater from both urban and cattle feedlot sources were collected over two years. Species identification of *Enterococcus* revealed distinct associations across the continuum. Of the 8430 isolates collected, *Enterococcus faecium* and *Enterococcus faecalis* were the main species in urban wastewater (90%) and clinical human isolates (99%); *Enterococcus hirae* predominated in cattle (92%) and feedlot catch-basins (60%), whereas natural streams harbored environmental *Enterococcus* spp. Whole-genome sequencing of *E. faecalis* (n = 366 isolates) and *E. faecium* (n = 342 isolates), revealed source clustering of isolates, indicative of distinct adaptation to their respective environments. Phenotypic resistance to tetracyclines and macrolides encoded by *tet(M)* and *erm(B)* respectively, was prevalent among *Enterococcus* spp. regardless of source. For *E. faecium* from cattle, resistance to β-lactams and quinolones was observed among 3% and 8% of isolates respectively, compared to 76% and 70% of human clinical isolates. Clinical vancomycin-resistant *E. faecium* exhibited high rates of multi-drug resistance, with resistance to all β-lactam, macrolides, and quinolones tested. Differences in the AMR profiles among isolates reflected antimicrobial use practices in each sector of the One-Health continuum.

## Introduction

Public concern for antimicrobial use (AMU) and resistance (AMR) in livestock is increasing, as is continuing pressure for industries and governments to address these concerns. Science-based and epidemiologically sound research is critical to drive policy, communication, legislation, and inform consumer choices. To effectively investigate the current state of antimicrobial resistance, holistic One Health approaches are required to determine correlation between AMU and AMR across the human-agriculture-environment continuum.

The genus *Enterococcus* is ubiquitous in nature and member species can be found in a range of habitats including soils, sediments, freshwater, marine water, beach sand, and a variety of plants^1,2^. *Enterococcus* spp. are also common members of the normal gastrointestinal (GI) flora of both livestock and humans^3^, with their concentrations in human and animal feces typically ranging from 10^3^–10^7^ cells per gram^4–6^*. Enterococcus* spp. are also commonly isolated from water contaminated by sewage or fecal wastes, and are widely used as bacteriological indicators of fecal contamination. However, “fecal” species have also been detected in various environmental samples with no obvious sources of contamination^1,7,8^ and likewise environmental *Enterococcus* spp. have been isolated from human and animal feces^9–11^. Some strains of fecal indicator bacteria (i.e., total coliforms, *Escherichia coli*, and *Enterococcus* spp.) can grow, multiply, and ultimately become naturalized to environments outside of the gastrointestinal tract^12^. *Enterococcus* spp. also exhibit greater environmental persistence than *E. coli* and are regarded as robust organisms capable of withstanding many environmental stressors^13–15^. Although considered commensals in humans, *Enterococcus* spp. are important opportunistic pathogens that not only form biofilms on catheters and implanted medical devices, but also cause urinary tract infections, bacteremia, endocarditis, burn and surgical site wound infections, abdomen and biliary infections^16^.

Foodborne *Enterococcus* spp. are rarely implicated as pathogens, but consumption of these bacteria can lead to their establishment in the GI tract^17^. The incidence of clinical infections caused by *Enterococcus* spp. has been steadily increasing since the 1970s^18–20^. *Enterococcus faecalis* and *Enterococcus faecium* are the most prevalent species in humans, and are implicated in the majority of healthcare-associated *Enterococcus* spp. infections^21^. Difficulties in treating *E. faecalis* and *E. faecium* have emerged due to acquired resistance, particularly multi-drug resistance to commonly used drugs including vancomycin.

*Enterococcus* spp. are intrinsically resistant to a number of antimicrobials including cephalosporins and trimethoprim-sulfamethoxazole, and exhibit low-level resistance to β-lactams and aminoglycosides. Clinical and animal *Enterococcus* isolates with multi-drug resistance to macrolides, tetracyclines, streptogramins, and glycopeptides have also been described^22,23^. Linezolid, daptomycin, and tigecycline may be used to treat serious invasive infections caused by vancomycin-resistant enterococci, with resistance to these antimicrobials mainly reported in clinical settings^18,24^. In contrast, tetracyclines and macrolides are used more frequently in livestock in North America^25,26^. Macrolides such as tilmicosin and tulathromycin are commonly administered to cattle to reduce or to treat bovine respiratory disease in North American feedlots^26^. In Europe, macrolides are widely used parenterally and orally for the treatment of common infections in food-producing animals^27^. In North America, the macrolide, tylosin phosphate is also included in beef cattle diets to prevent liver abscesses^26^, a practice that has been discontinued in Europe. Previous research has demonstrated that therapeutic and subtherapeutic administrations of macrolides to cattle increase the proportion of erythromycin resistant enterococci in bovine feces^28^. Modern farming practices also commonly add metals such as zinc and copper to livestock feed to ensure nutritional requirements are met. Linkage of metal resistance to AMR such as methicillin in staphylococci, or macrolides and glycopeptides in enterococci has also been reported^29^.

The World Health Organization and Health Canada categorize antimicrobials according to their importance to human health. Macrolides, belonging to the drug superfamily MLSB (macrolide–lincosamide–streptogramin B) are commonly used as a first line treatment in humans, and for disease prevention and control in food animals, including beef cattle. Residues of veterinary antimicrobials in drainage runoff from confined feeding operations (feedlots), manure storage facilities, or manure-amended crop and pasture land are also a potential cause for concern, especially if the runoff enters receiving waters. Where present, residues in beef cattle manure could promote AMR, thus compromising environmental health, and potentially the efficacy of antimicrobials in veterinary and human medicine. There are three principal zoonotic transmission routes of AMR bacteria from agri-food systems to humans: (1) the consumption of contaminated food; (2) direct contact between humans and livestock and (3) environmental dispersion. Horizontal gene transfer can lead to genetic exchange of antimicrobial resistance genes (ARGs) among bacteria, facilitating the dissemination of AMR, so that both bacteria and ARGs should be considered in surveillance systems. Commensal bacteria that link different arenas of the biosphere also have important roles in ARG dissemination^30^. Thus, ARG-carrying *Enterococcus* spp. may transmit ARGs and cause infections in humans. The objectives of this research were to determine the extent to which beef cattle may contribute to AMR of indicator bacteria from feedlot and downstream environmental reservoirs relative to AMR in the public, and to characterize the potential chain of transmission to humans.

## Results

### Sampling feedlot and downstream environment, a beef processing plant, retail meat and urban wastewater

**Figure 1:**
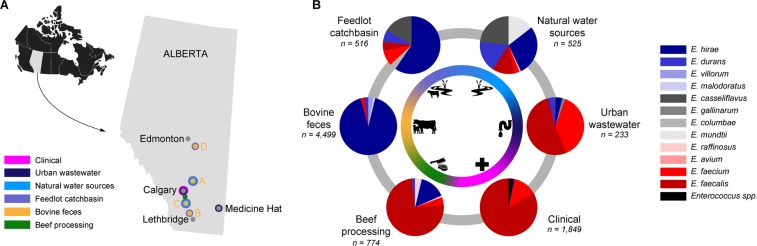
(**A**) Sampling locations in the province of Alberta, Canada, for the isolation of *Enterococcus* spp. (**B**) Proportion of *Enterococcus* species isolated from beef processing (abattoir and retail meat), bovine feces, feedlot catch basin, surface water (natural water resources), urban wastewater and human clinical isolates (Calgary Laboratory Services, Calgary, **AB**), including both selective (erythromycin-resistance) and non-selective isolates, as determined by sequencing of the *groES – groEL* intergenic region and comparison against the *Enterococcus* spp. *groES-EL* intergenic region custom database.

Two years of sampling was completed in April 2016, collecting a total of 1,728 samples (Supplementary File 1, Table S1). Composite fecal pen samples collected from four feedlots (A, B, C, D) at varying locations in Alberta (Fig. 1A) comprised the majority of samples (n = 866). Catch basins associated with all four feedlots provided 72 samples, whereas 57 surface water samples were collected near feedlots A and C. Soil collected from two agricultural fields (East field and West field) adjacent to Feedlot C constituted 25 samples, whereas 5 manure samples were collected from stockpiled manure at East field (Supplementary File 1, Tables S1 and S2). Urban wastewater contributed 31 samples in total including composite influent (post-grit tank; n = 22) and effluent (immediately prior to release; n = 21), which were collected over the same two year period as the fecal samples. A total of 600 samples were collected from a beef processing plant over 9 sampling trips over a one year period (April, 2015 – March 2016). At the same time, 60 retail meat samples were collected from local super markets within the sampling region (Supplementary File 1, Table S1).

### Enterococcus recovery

A total of 8,307 presumptive *Enterococcus* spp. isolates were recovered from all sites/sources tested including bovine feces (n = 4,499), feedlot catch basins (n = 510), surface water/natural water sources (n = 521), urban wastewater influent and effluent (n = 222), beef processing (abattoir and retail beef) (n = 774), and human clinical cases (n = 1,849) (Fig. 1B). The majority of feedlot samples were positive for *Enterococcus* spp. (average 95.0%), with both non-selective and selective isolate recovery regimes (Supplementary File 1, Table S2). Recovery from environmental sampling locations was highly variable, ranging from no recovery (soil samples), to 100% (e.g., downstream ephemeral creek site) (Supplementary File 1, Table S2). The rate of isolation from selective and non-selective plates was often similar between beef fecal samples and other sources rich in fecal matter (e.g., catch basin, wastewater influent). Isolate recovery rates from surface water, wastewater effluent and meat processing samples was lower for antibiotic-free as compared to non-selective media (Supplementary File 1, Table S2).

### Enterococcus species diversity

*Enterococcus hirae* was the predominant species recovered from cattle production systems including both bovine feces (92%), and feedlot catch basins (60%) (Fig. 1B; Supplementary File 1, Table S3). Other *Enterococcus* spp. found in bovine feces included *E. villorum*, *E. durans*, *E. faecium*, *E. malodoratus*, *E. faecalis*, *E. casseliflavus*, *E. mundtii*, *E. avium*, *E. columbae*, and *E. gallinarum* (Supplementary File 1, Table S3). *Enterococcus hirae* (71%) and *E. casseliflavus* (29%) were the only two species isolated from stockpiled manure. *Enterococcus* species recovered from catch basin wastewater included *E. casseliflavus* (17%), *E. faecium* (9%), *E. durans* (7%), *E. faecalis* (5%), *E. gallinarum* (4%), and *E. mundti* (0.2%). Samples from the abattoir and retail beef had a high abundance of *E. faecalis* (74%) followed by *E. hirae* (15%) and *E. faecium* (4%). A wide variety of other *Enterococcus* spp. were also recovered from beef processing samples, including, *E. raffinosus*, *E. durans*, *E. malodoratus*, *E. gallinarum*, *E. casseliflavus*, *E. mundtii*, and *E. avium* (Supplementary File 1, Table S3). Species diversity (10 different *Enterococcus* spp.) in beef processing isolates was second only to bovine feces, where 11 different *Enterococcus* spp. were recovered.

Enterococci recovered from surface water sources in the vicinity of feedlot C included *E. hirae* (28%), *E. casseliflavus* (23%), *E. durans* (17%), *E. mundtii* (14%), *E. faecium* (10%), *E. faecalis* (8%), and *E. gallinarum* (0.76%) (Table S3). The majority of *E. hirae* isolated from water samples originated from the wetland and ephemeral creek downstream of the feedlot. *E. faecalis* (52%) and *E. faecium* (38%) were the most prevalent species in urban wastewater, whereas *E. durans*, *E. hirae*, *E. gallinarum* and *E. casseliflavus* were less abundant (0.4–4%).

A total of 1,849 individual human clinical *Enterococcus spp*. obtained from Calgary Laboratory Services (CLS) were studied. Most of these were isolated from non-sterile site (NSS) specimens (n = 1,644; 89%) including urine, wounds, catheters etc.). Forty-three (2%) of sterile site (SS) isolates were recovered from blood and other fluids and tissues, while 162 (9%) vancomycin resistant enterococci (VRE) were mostly recovered from rectal surveillance swabs from hospitalized patients, but a few also came from NSS/SS specimens including blood, urine and abdominal abscess drainage. Of NSS isolates, 90% were identified as *E. faecalis*, and 5% as *E. faecium*, while only 3 isolates were identified as *E. hirae*, *E. casseliflavus* and *E. gallolyticus*. A species-level identification was not initially obtained for 52 (3%) isolates in the NSS category using MALDI-TOF MS, and these were clinically reported as *Enterococcus* spp. by CLS. Fifty of these isolates were later characterized via whole-genome sequencing. Sterile category (SS) isolates included *E. faecalis* (n = 32), *E. faecium* (n = 8), *E. casseliflavus* (n = 2) and *E. gallinarum* (n = 1). Most VRE isolates (159; 98%) were identified as *E. faecium*, and only three were *E. faecalis*.

### Antimicrobial resistance phenotyping

**Figure 2:**
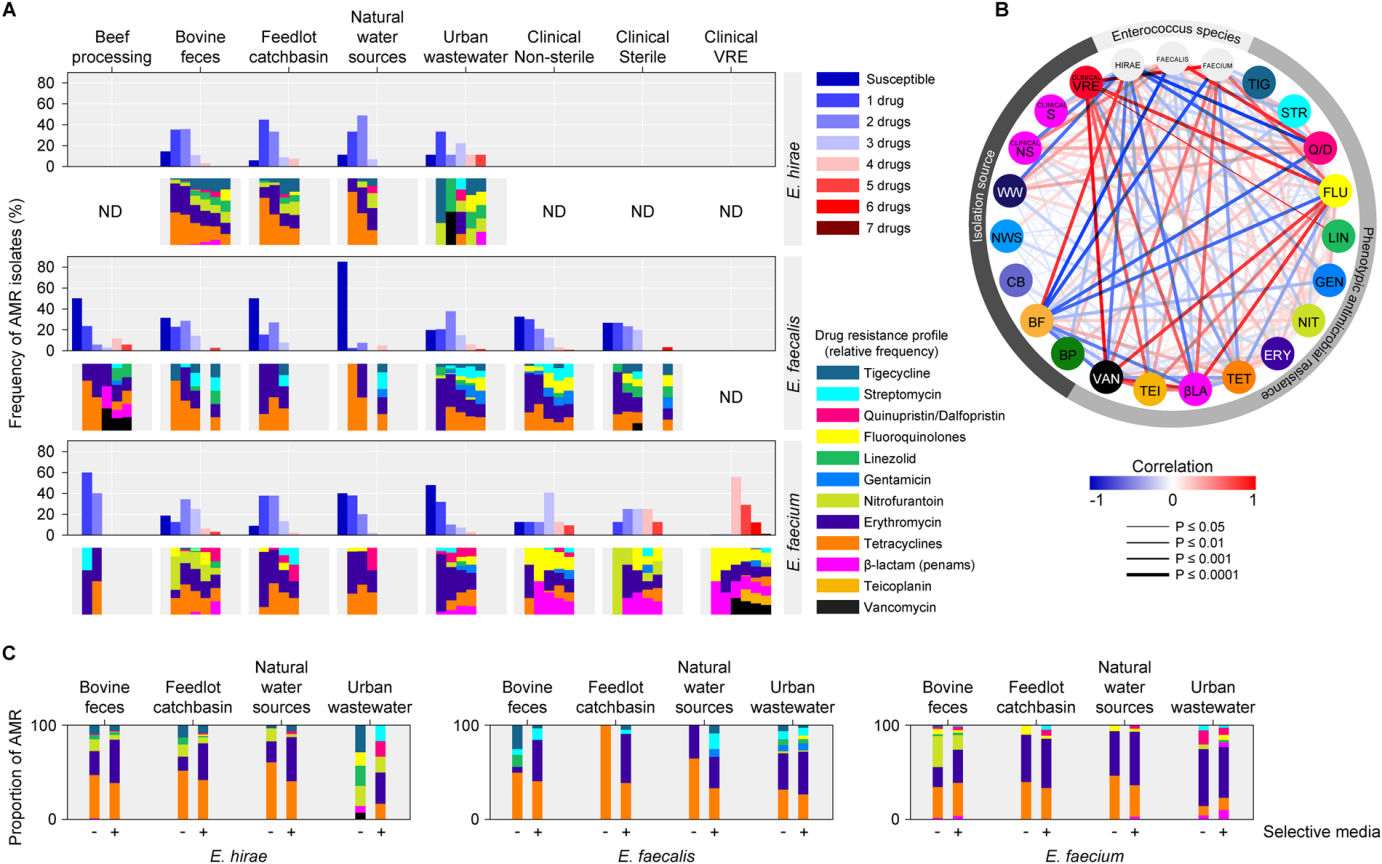
(**A**) Overall frequency of AMR phenotypes in enterococci species isolates from across the one-health continuum. Upper panels for *E. hirae*, *E. faecalis* and *E. faecium* indicate prevalence of resistance to various numbers of drugs, while lower panels indicate drug resistance class profiles; (**B**) Correlation between isolation sources, *Enterococcus* species and antimicrobial resistance phenotypes (a measure of association was determined through Pearson’s correlation analysis); (**C**) Comparison of proportion of phenotypic AMR between enterococci isolated on antibiotic-free and macrolide (erythromycin) selective media. BP, beef processing (abattoir & retail meat); BF, bovine feces; CB, catch basin; NWS, natural water source; WW, waste water; clinical NS, clinical non-sterile; clinical S, clinical sterile; clinical VRE, clinical vancomycin resistant. ND (not detected/not determined/no data) indicates lack of data-points due to the absence or low detection of a particular *Enterococcus* species in a sector of continuum.

Tetracycline and macrolide resistance were among the most prevalent phenotypes in *E. hirae*, *E. faecalis*, and *E. faecium* collected from feedlot and downstream environments (Fig. 2A,C), and were frequently positively correlated (Fig. 2B; p = ≤0.001). These two phenotypes were also abundant in *E. faecalis* and *E. faecium* from urban wastewater as well as in *E. faecalis* from human clinical samples.

Tetracycline resistance was most abundant in bovine fecal *E. hirae* isolated on antibiotic-free media (59%) followed by macrolide resistance (33%), nitrofurantoin (16%), tigecycline (12%), linozelid (6%), β-lactam (ampicillin; 1.5%), and quinopristin/dalfopristin (1.4%; Fig. 2A). Similarly, catch basin and surface water *E. hirae* had a high prevalence of tetracycline (72% and 79%, respectively) and macrolide resistance (21% and 29%, respectively). The majority of *E. hirae* isolated on BEA-Ery selective media originated from bovine feces (68%), catch basin (84%), and surface water (76%) were resistant to tetracycline (Fig. 2C; Supplementary File 2). Multidrug resistance (MDR; resistance to ≥2 drugs) was present in 37%, 33%, and 39% of *E. hirae* isolated on antibiotic-free media from bovine feces, catch basin, and surface water, respectively.

Among *E. faecalis* isolates from bovine feces isolated on antibiotic-free media, tetracycline resistance was most prevalent (38%), followed by tigecycline (19%), linezolid (10%) and macrolide (5%) resistance, whereas 93% of bovine fecal *E. faecalis* isolated on BEA-Ery media were resistant to tetracycline (Fig. 2C). For *E. faecium* recovered on antibiotic-free media from feedlots, resistance to tetracycline and nitrofurantoin was most abundant (45% for each), followed by macrolide (29%), and fluoroquinolone (8%) resistance. Other low abundance resistance phenotypes were present at ~3% (i.e., resistance to β-lactam, linezolid, tigecycline and quinopristin/dalfopristin) (Supplementary File 2). All of the macrolide-resistant *E. faecium* isolated from BEA-Ery selective media were resistant to tetracycline.

*E. faecalis* isolated from NSS compared to SS clinical specimens showed that 32% and 51%, respectively, were resistant to both macrolide and tetracycline drugs, whereas only 24% of *E. faecalis* isolates from wastewater demonstrated this resistance profile. Occurrence of *E. faecium* resistance to β-lactams (76%) and quinolones (70%) was also much higher in isolates from clinical specimens than those from feedlots (3% and 8%, respectively). Clinical *E. faecium* isolates also showed high prevalence of resistance to macrolides (68%) and tetracyclines (24%). VRE isolates were also multi-drug resistant to macrolide, β-lactam, and quinolone drugs and resistance to teicoplanin, tetracycline and quinopristin/dalfopristin was depicted by 40%, 8% and 0.7% of VRE, respectively. High-level aminoglycoside resistance was also found in 8% of VRE.

For *E. faecalis*, 5% (bovine feces), 3% (surface water), and 28% (urban wastewater) of isolates recovered from antibiotic-free media were MDR compared to clinical *E. faecalis* isolates which had a higher rate of MDR (36%). None of the *E. faecalis* isolated on antibiotic-free media from catch basins were MDR. Among *E. faecium*, 47% (bovine feces), 17% (catch basin), 6% (surface water), and 9% (urban wastewater) of isolates from antibiotic-free media were MDR compared to clinical non-VRE and VRE isolates that had an MDR rate of 78% and 100%, respectively. Overall, erythromycin and tetracycline were the most common resistance phenotypes in *E. faecalis* across all sample sources. Urban wastewater and human clinical isolates had a greater diversity of resistance phenotypes and harboured higher levels of multidrug resistance as compared to feedlot and downstream *E. faecalis*.

Except for the clinical VRE isolates, no *E. faecium* isolated from other sources were resistant to vancomycin. Among *E. faecalis*, only 2/153 human clinical isolates were resistant to vancomycin. Only one *E. hirae* from bovine feces was resistant to both vancomycin and teicoplanin, while another *E. hirae* isolate from urban wastewater was resistant to vancomycin.

### Whole genome sequence analyses

Whole-genome sequencing (WGS) was conducted on 366 *E. faecalis* and 342 *E. faecium* isolates selected form across the One-Health continuum. Amongst all clinical isolates analyzed via WGS (n = 299), 50 were included from the NSS category that could not be previously identified to the species-level at the CLS facility using non-molecular methods. These isolates were identified by WGS as *E. faecalis* (n = 42), *E. faecium* (n = 5), *E. casseliflavus* (n = 1), *E. hirae* (n = 1), and *E. raffinosis* (n = 1). The WGS data of all 708 isolates were further characterized for the presence of ARGs, virulence genes, phylogenomic relatedness, MLST, and mobilome.

#### Phylogenomics and MLST

**Figure 3:**
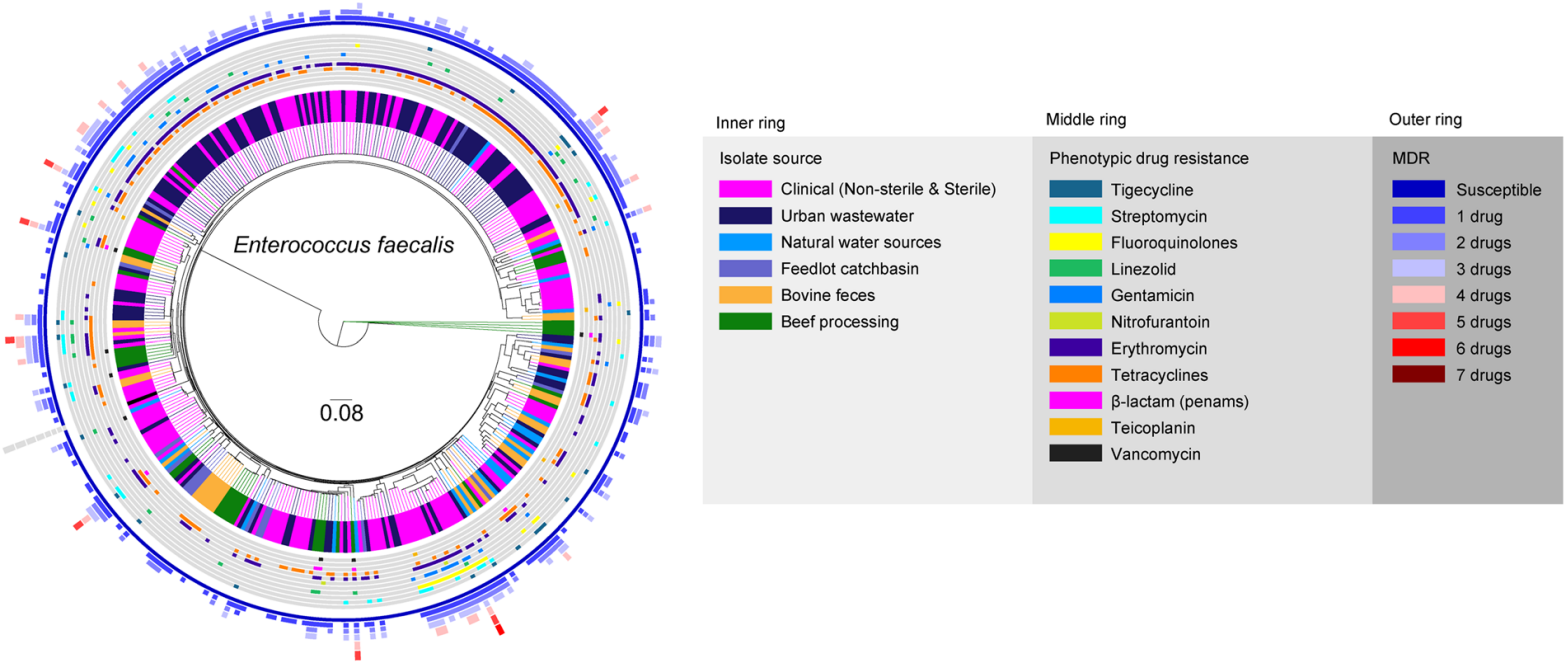
Phylogenomic relatedness tree constructed based on analysis of single-nucleotide polymorphisms (SNPs) of the core genes of *E. faecalis* genomes (n = 366) isolated from various environments and samples related to beef production and processing systems, environment, and human-related sources. The genomes were compared using *E. faecalis* OG1RF genome (GenBank accession # NC_004668/CP002621.1) as a reference. Phylogenomic analysis of the WGS data was conducted using SNVPhyl^87^ and the resulting newick tree file was visualized in FigTree v1.4.4 (http://tree.bio.ed.ac.uk/software/figtree/) to obtain phylogenomic dendrogram.

Phylogenomic analyses of *E. faecalis* and *E. faecium* revealed their high genomic diversity, with 62% and 45% of their genomes constituting the core genome, respectively. The majority of beef production and human-related isolates were not closely related as indicated by their differential phylogenomic clustering and MLST types. In contrast, the majority of urban wastewater and human clinical *E. faecalis* isolates were closely related. Most of the wastewater (62%) and a large proportion of the human isolates (34%) clustered into a single clade (Fig. 3). Other small clades were formed by *E. faecalis* originating from wastewater and human sources, occasionally including isolates from other sources. *E. faecalis* isolates from bovine feces, catch-basins, and surface water in the vicinity of feedlots were more diverse than clinical and urban wastewater isolates and formed mixed clusters across the tree. Feedlot isolate genomes were more closely related to catch basin and surface water derived *E. faecalis*. The beef-processing isolates originating from abattoir and retail meat samples were also diverse. Four of the beef-processing isolates including three isolates from the abattoir and one from retail beef formed an unique clade, and another 6 retail isolates clustered as a sub-clade—within a clade that included a sub-clade exclusively comprised of bovine fecal isolates. Other beef-processing isolates contributed to multiple mixed clusters.

**Figure 4:**
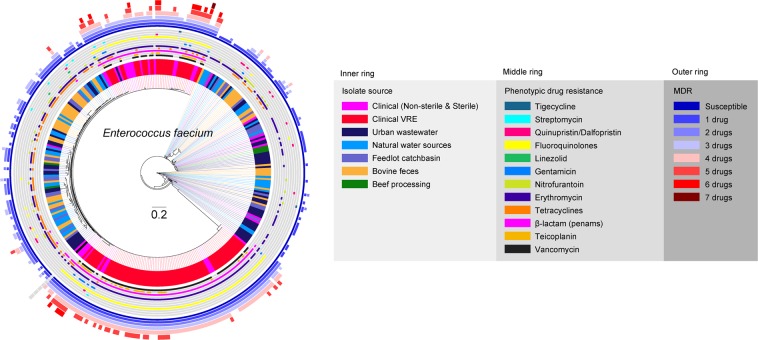
Phylogenomic relatedness tree constructed based on analysis of single-nucleotide polymorphisms (SNPs) of the core genes of *E. faecium* genomes (n = 342) isolated from various environments and samples related to beef production and processing systems, environment, and human-related sources. The genomes were compared using *E. faecium* DO genome (GenBank accession# CP003583.1) as a reference. Phylogenomic analysis of the WGS data was conducted using SNVPhyl^87^ and the resulting newick tree file was visualized in FigTree v1.4.4 (http://tree.bio.ed.ac.uk/software/figtree/) to obtain phylogenomic dendrogram.

For *E. faecium*, two large clusters were formed predominantly by human VRE along with some non-VRE human isolates (Fig. 4). Human isolates also frequently contributed to clades that included wastewater isolates. The majority of feedlot and downstream environmental isolates of *E. faecium* formed mixed clades indicating their genomic similarities. Occasionally, surface water isolates co-clustered with wastewater isolates. Based on lower shared core genome and variable clustering, a higher genomic diversity was discovered for *E. faecium* compared to *E. faecalis* across the continuum, with occasional co-clustering of beef production and human related isolates.

Whole-genome sequence data was subjected to multi-locus sequence typing (MLST) using PubMLST^31^. Similar to their diverse phylogenies, both *E. faecalis* and *E. faecium* exhibited a high diversity of sequence type (ST) groups (Supplementary File 1, Figure S1; Supplementary File 3). Of the 366 *E. faecalis*, 85% of the isolates (n = 311) belonged to 66 known STs, but no ST could be assigned for remaining 15% (n = 55). Twenty-one percent of bovine fecal *E. faecalis* were ST86; 9% were ST21; 51% belonged to twelve other ST groups, and 18% had unknown ST assignments. *E. faecalis* with ST21 were also isolated from beef processing (12%), urban wastewater (3%), and human clinical (3.4%) sources. For human clinical *E. faecalis*, ST179 was most prevalent (26%) among known ST groups followed by ST40 (7%), ST64 (4%), and ST778 (4%), with 45% belonging to thirty other ST groups and 12% with unknown ST assignments. As with *E. faecalis* clinical isolates, ST179 was most prevalent (38%) among urban wastewater isolates, followed by ST16 (20%) (Supplementary File 1, Figure S1, and Supplementary File 3).

Of the 342 *E. faecium* tested, 73% isolates (n = 250) were associated with 62 known ST groups while the rest (n = 92) had unknown ST groups. Among bovine fecal *E. faecium*, 49% of isolates were assigned to unknown ST, while the most prevalent known STs were ST32 (9%) and ST29 (7%), with 20% of the isolates belonging to 15 other known ST groups. Among feedlot catch basin isolates, 60% of isolates had unknown ST whereas ST94 (14%) was the most abundant known ST, and was shared by isolates from other sources including surface water (4.4%), urban wastewater (11%) and the clinic (5.4%). ST117 was most abundant among clinical VRE (76%) and non-VRE (35%) *E. faecium*, whereas the closely related ST 17 was detected at 0.9% and 11% within VRE and non-VRE isolates.

#### Genetic determinants of AMR

**Figure 5:**
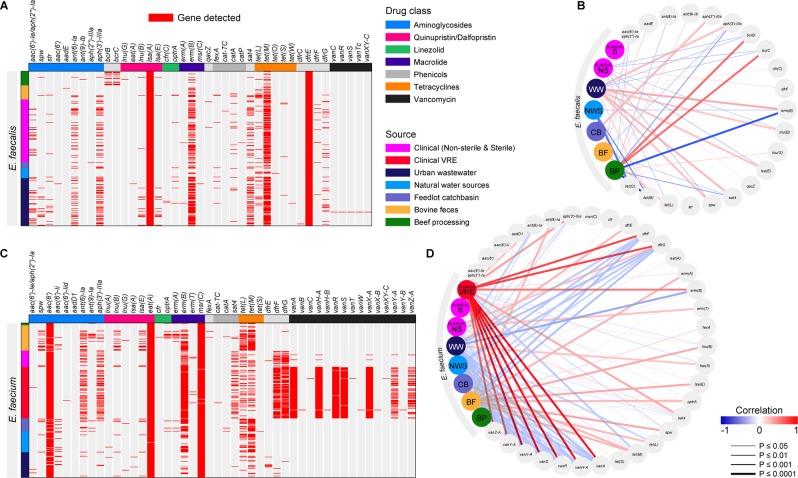
Detection of antimicrobial resistance genes (ARGs) in whole genome sequenced *E. faecalis* (**A**) and *E. faecium* (**C**) and their correlation plots respectively (**B**,**D**) to demonstrate association between the isolate sources and ARGs. A measure of association was determined through Pearson’s correlation analysis.

Using the NCBI AMR gene database, WGS data was mined for the presence of ARGs in *E. faecalis* (n = 366) and *E. faecium* (n = 342). Across all isolates, 41 ARGs were associated with *E. faecalis*, and 46 with *E. faecium*, with 30 ARGs shared between the two species (Fig. 5A,C, and Supplementary File 4). Total ARGs (n = 56; Supplementary Table S4) collectively associated with both species conferred resistance to a variety of antimicrobials including: aminoglycoside, quaternary ammonium compound small multidrug resistance (SMR) family of drug efflux pumps, linezolid, phenicol, oxazolidinone-phenicol transferable resistance, macrolide, lincosamide, streptogramin A, pleuromutilins, streptothricin, tetracycline, trimethoprim and vancomycin.

Sequencing data indicated that all *E. faecalis* isolates harboured *dfrE* and *lsa(A)* genes, whereas all *E. faecium* had *aac(6*′*)* and *eat(A)* genes. Across all sample sources, *erm(B)* and *tet(M)* were frequently present in both *E. faecalis* and *E. faecium*. Aminoglycoside ARGs, including *ant(6)-Ia*, *aph(3*′*)-IIIa*, and *sat4*, and tetracycline ARGs *tet*(L) and tet*(M)* were detected among *E. faecalis* from all sample sources. Tetracycline ARGs *tet(L)* and *tet(M)* were also detected in *E. faecium* isolated from all sources. Compared to other sources, the ARGs *ant(6)-Ia*, *sat4*, and *aph(3*′*)-IIIa*, that code for resistance to streptomycin, streptothricin, and kanamycin respectively, were detected at high frequencies in *E. faecalis* from wastewater (25%, 42%, 42%), as well as in clinical *E. faecalis* (29%, 23%, and 26%) and *E. faecium* (40%, 33%, and 43%). The *optrA* gene was only present in livestock associated *E. faecium* sequenced from fecal (14%), catch basin (10%), and beef processing (3%) sources, whereas in *E. faecalis* this gene was present at low frequency (1.6%) across the continuum and appeared to be linked to the phenicol resistance *fexA* gene. The quaternary ammonium compound efflux SMR genes *bcrB* and *bcrC* were frequently (35%) found in *E. faecalis* isolates from the beef processing plant (Fig. 5B). Trimethoprim resistance genes *dfrF* and *dfrG* were detected (up to 83%) in human clinical *E. faecium*, and less frequently in wastewater isolates (up to 5%). All but two genes (*vanY* and *vanZ*) of the *vanA* vancomycin resistance cluster were always present in all VRE isolates, while *vanY-Z* were detected in 50% of VRE. Wastewater *E. faecalis* frequently possessed a number of ARGs conferring resistance to aminoglycoside, macrolide, lincosamide and streptogramin A and tetracycline, whereas bovine isolates were more frequently associated with tetracycline ARGs (Fig. 5B). Among *E. faecium*, clinical VREs also frequently harboured multiple ARGs coding for resistance to aminoglycoside, macrolide, trimethoprim, and vancomycin, while bovine isolates were more often carrying aminoglycoside, macrolide, lincosamide and streptogramin A, oxazolidinone, and tetracycline ARGs (Fig. 5D).

#### Virulence genes

Assembled genomes of *Enterococcus* spp. were assessed for virulence genes contained in the virulence factor database (VFDB); Supplementary File 4). For *E. faecalis*, 49 different virulence genes were identified, including genes coding for adhesins, aggregation substances, hyaluronidases, biofilm-associated proteins, lipopolysaccharide biosynthesis proteins, bile salt hydrolase, caseinolytic protease, capsular polysaccharide biosynthesis, cytolysin production, collagen binding protein, endocarditis specific antigen, virulence regulator/signal transduction system, fibrinogen adhesins, fimbrial biogenesis, metal binding lipoprotein, and serine protease (Supplementary File 1 Table S5). Eleven of these genes (ace, *bopD*, *ebpA*, *ebpB*, *ebpC*, *srtC*; *clpP*; *cpsA*, *cpsB*; *efaA*; *fss1*) coding for biofilm-associated proteins, caseinolytic protease, capsular polysaccharide biosynthesis, endocarditis specific antigen and fibrinogen adhesins were found in all *E. faecalis*, whereas *hylA*, *gelE*, and *sprE* coding for hyaluronidases, biofilm-associated proteins and serine protease respectively, were present in >86% of all isolates from this species. Eight cytolysin production genes were predominantly associated with clinical (54%) and urban wastewater (63%) *E. faecalis*, and were rarely present in isolates from other sources. Only 1/34 beef processing *E. faecalis* isolates carried cytolysin genes. The fibrinogen adhesion gene *fss3* was also abundant in clinical (58%) and urban wastewater (47%) isolates. Of 34 *E. faecalis* beef processing isolates, 14 originated from retail beef; and 6/14 isolates (43%) carried the *fss3* gene. This gene was absent in bovine fecal isolates and was present in 1 isolate from the catch basin, and 1 isolate from surface water.

In *E. faecium* genomes, 33 virulence genes, related to the above described virulence factor categories were found. Of these, only two biofilm-associated protein and caseinolytic protease coding genes *bopD* and *clpP*, were present in all *E. faecalis*, while four genes related to collagen binding protein (*acm*, *scm*), bile salt hydrolase (*bsh*), and adhesion (*sgrA*) were found in 92%, 75%, 71%, and 64%, respectively, of all *E. faecium*. Three genes *fss3* (77%), *ecbA* (72%), and *psaA* (71%), coding for fibrinogen adhesion, adhesins and metal binding lipoprotein respectively, were predominant in clinical *E. faecalis*, and were absent or rarely present in isolates from other sources. Thirty of these genes were shared between the two *Enterococcus* species

#### Plasmids

Across the One Health continuum, 28 different plasmid types were identified in *E. faecalis*, while 23 were found in *E. faecium* (Supplementary File 1, Figures S2 and S3; Supplementary File 4). For *E. faecalis*, unique plasmid profiles could be identified among various sample types, with beef processing/retail samples having p703/5 (59%), pEJ97-1 (50%), pEF1071 (44%), and pAD1 (38%). Plasmid pTEF2 was the most prevalent type in clinical (52%) and urban wastewater (49%). The plasmid pAD1 was prevalent in clinical *E. faecalis* (30%); p703/5 t was prevalent in urban wastewater (24%), and pTEF2 was frequent in surface water isolates (32%). Bovine fecal and catch basin *E. faecalis* harboured 14 and 12 different plasmids, respectively, with no obvious differences in plasmid abundance. For *E. faecium*, in addition to uncharacterized plasmid contigs of *E. faecium* strain 287, clinical isolates had the highest abundance of pB82 (78%) followed by p703/5 (72%), pEFNP1 (45%) and pRUM (34%). Plasmid pAMbeta1 was present in *E. faecium* from all sample types including wastewater (39%), catch basin water (31%), surface water (26%), and bovine feces (18%), but was only found in 2/150 sequenced clinical *E. faecium*.

## Discussion

Throughout the continuum, a clear delineation of species present in different environments was observed with *E. faecium* and *E. faecalis* being the predominant species associated with humans (clinic and urban wastewater), while *E. hirae* was the predominant species isolated from cattle feces and associated wastewaters. A shift in the predominant cattle associated *Enterococcus species* to more naturalized species in the environmental samples indicates limited/no evidence to support transmission across the continuum. These findings are relevant to the investigation of AMR transmission as they help delineate the extent of microbial transmission along the agricultural-environmental-public continuum, and suggest that the major species within each sample type have niche-specific adaptations. Consistent with our findings, *E. casseliflavus* and *E. mundti* are most often associated with vegetation and forage crops^1,32^, whereas *E. hirae* is the most abundant *Enterococcus* species in bovine feces^28,33,34^. The species differences between bovine and human clinical isolates combined with differential phylogenomic clustering suggest that *Enterococcus* spp. from beef cattle may not present a significant source or reservoir of human-associated *Enterococcus* spp. (i.e., *E. faecium* and *E. faecalis*). These findings also suggest that dissemination of bovine-associated *Enterococcus* spp. (i.e., *E. hirae*) beyond feedlots is minimal. Although *E. hirae* was predominantly associated with cattle, its overall relative prevalence in beef processing system/abattoir/retail meat was ~15%, whereas *E. faecalis* was most abundant (74%). In general, the prevalence of *E. hirae*, as well as other *Enterococcus* spp. on carcasses decreased along the processing chain (data not shown). However, a two-fold increase in species distribution of *E. faecium* and *E. faecalis* (42–80%) between carcass and retail meat could be a reflection of downstream contamination of meat products by isolates originating from humans. Nonetheless, the beef-processing isolates investigated in this study were phylogenomically diverse and co-clustered with multiple sample types including animal, human clinical and urban wastewater. Overall, *E. faecalis* had a higher recovery rate than *E. faecium* from beef processing/retail meat, urban wastewater, and clinical samples (Fig. 1B), which may be related to its prevalence in the intestinal tract of humans^20,35^ and its greater persistence within the gut than *E. faecium*^36^. The higher population density of *E. faecalis* relative to *E. faecium*, in combination with its broader host-range, could also provide *E. faecalis* with more opportunities to exchange genetic material than *E. faecium*.

Although *E. hirae*, as well as other *Enterococcus* spp. (i.e., *E. avium*, *E. durans*, *E. casseliflavus*, *E. gallinarum*, and *E. raffinosus*) can cause clinical infections in humans, such cases are rare and considered opportunistic^37,38^. To our knowledge, there have only been 10 case reports of *E. hirae* causing septicemia, endocarditis, spondylodiscitis, pyelonephritis, cholangitis, peritonitis, or urinary tract infections in humans^39^. In comparison, *E. faecalis* and *E. faecium* are the most common species implicated and are responsible for as much as a third of the nosocomial infections worldwide^40^. The recent emergence of *E. faecium* and *E. faecalis* as MDR nosocomial pathogens appears to be driven by the ubiquitous nature of *Enterococcus* spp., and other factors, such as the plasticity of their genomes, and the widespread use of antibiotics^20^.

Overall, resistance to tetracycline and macrolides appears to be ubiquitous in all three of the major *Enterococcus* spp. recovered (i.e., *E. faecalis*, *E. faecium* and *E. hirae*), likely reflecting the widespread use of these antibiotics in both human and veterinary medicine. Considering the importance of *E. faecalis* and *E. faecium* in nosocomial infections, we further investigated these two species at the genomic level to identify and compare their virulence and ARGs. ARGs found in 708 *E. faecalis* and *E. faecium* isolates sequenced in this study corroborated phenotypic AST data and highlighted the ubiquitous nature of tetracycline (*tet L, tet M)* and macrolide (*erm(B)*) ARGs in both species. Both *erm(B)* and *tet(M)* were often associated with the transposon Tn1545^41,42^, likely increasing the frequency of horizontal transfer of these ARGs among *Enterococcus* spp.^43^. In *Enterococcus* spp. *erm(B)*, a ribosomal methylase has been considered as the most prevalent gene conferring resistance to erythromycin^44^. In addition to drug target modification by ribosomal methylation, resistance to macrolides can also arise because of mutations in the 23S rRNA gene or by efflux pumps^45^. In our study, with the exception of *msr(C)* in *E. faecium*, (which exhibited no associated macrolide resistant phenotype), no other efflux-encoding genes were identified. Previous studies have identified *erm(B)* in *E. hirae*^11^ from cattle, but found *tet(O)* instead of *tet(M)*, which encode for ribosomal protection proteins^46^. Considering that most *E. hirae* identified were resistant to tetracycline and macrolides, future comparative genomic analyses are needed to determine the genetic similarity of their respective ARGs to those in *E. faecalis* and *E. faecium*.

All *E. faecalis* isolates harboured *dfrE* and *lsa(A)* genes, consistent with the fact that *E. faecalis* intrinsically carry *dfrE* despite being susceptible to trimethoprim^47^. This species also carries *lsa(A)* that codes for intrinsic resistance to clindamycin and quinupristin-dalfopristin (Q/D)^48^. Consistent with the genotype, the phenotypic antimicrobial susceptibility data indicated that all *E. faecalis* were resistant to quinupristin-dalfopristin, but clindamycin and trimethoprim susceptibilities were not tested in our study. All sequenced *E. faecium* carried *aac(6*′*)*, *eat(A)*, and *msr(C)* genes in their genomes. All of these genes have been previously reported to be present in *E. faecium* and appear to be specific to this species. *Enterococcus* spp. exhibit intrinsic resistance to aminoglycosides as a result of the presence of aac(6′)^49^. The *eat(A)* gene product shows similarity with Lsa(A), Lsa(E), Lsa(B), and Lsa(C) proteins that confer lincosamides, streptogramins A, and pleuromutilins (LS_A_P) resistance in various Gram-positive bacteria, and is speculated to be involved in protein translation^50^. Similarly, *E. faecium msr(C)* had similar properties to other macrolide resistance proteins of the MsrA and MsrC categories, suggesting it may also mediate efflux and be a remote progenitor of the acquired macrolide resistance gene^49–51^. Furthermore, as these genes appear to be species specific, they have the potential to aide in species identification.

Compared to other sources, human associated (clinical and urban wastewater) *Enterococcus* spp. frequently harbored ARGs encoding resistance to streptomycin, streptothricin, and kanamycin, commonly found as a cluster (*aadE/ant(6)-la– sat4– aph(3*′*)-IIIa*) that is generally associated with insertion elements from Tn5405 transposons. This cluster has been described in *Staphylococcus, Streptococcus* and *Enterococcus* spp.^34,52–54^. Although this gene cluster is generally part of a Tn5405 transposon, a high level of heterogeneity of Tn5405-related elements with different arrangements, including deletions and insertions of ORFs, has been described in both *Enterococcus* and *Staphylococcus* spp.^52,55^. This gene cluster has also been identified in Tn1545 from *Streptococcus pneumoniae*^56^, suggesting a widespread occurrence among Gram-positive bacteria. Linkage between this gene cluster and *erm(B)* has also been described in *E. faecium* and *Staphylococcus intermedius*^55,57^. The rare occurrence of this cluster in feedlot and environmental *E. faecalis* and *E. faecium* suggests limited transfer of these genes across the One-Health continuum. The abundance of this element exclusively in human-associated *Enterococcus* spp. is likely a reflection of the use of these associated antimicrobials in humans.

The *optrA* gene, encoding for an ABC transporter, was only detected in livestock associated *E. faecium* isolated from beef processing, bovine fecal and catch basins. Plasmid-borne *optrA* is known to confer transferable resistance to oxazolidinones (linezolid and tedizolid) and phenicols in *E. faecalis* and *E. faecium*. Previous studies^58^ also detected this gene more frequently in *E. faecalis* and *E. faecium* from food-producing animals than from humans. This gene is often associated with the conjugative plasmid, pE349 alongside a phenicol exporter gene, *fexA*. The *optrA* gene is also frequently surrounded by insertion sequences, regardless of whether it is associated with the chromosome or plasmids^59,60^. In our study, a linkage between *optrA – fexA* was apparent in *E. faecalis*, but not in *E. faecium*, suggesting difference in gene acquisition by these species.

Except for the clinical *E. faecium* VREs, vancomycin resistance was found sporadically in *E. faecalis* and *E. hirae*. However, no ARGs linked to this resistance phenotype were identified in *E. faecalis*. Previous studies have also identified vancomycin resistance in this species that could not be attributed to known resistance genes^61^. Since we generated no sequence data for *E. hirae* in this study the identity of vancomycin ARGs in the two resistant isolates of this species is unknown. There are a variety of known glycopeptide resistance operons, including: *vanA*, *vanB*, *vanC*, *vanD*, *vanE*, *vanG*, *vanL*, *vanM*. and *vanN*. Of these*, van*A is the most common and is carried by the Tn3-family transposon Tn1546 in clinical settings^16^. Current data also indicates the presence of all *vanA* operon genes in all clinical *E. faecium* VREs. All but two genes, *vanY* and *vanZ* of the *vanA* vancomycin resistance cluster were present in all clinical *E. faecium* VRE isolates, while *vanZ* was detected in ~46% of these VREs. The most common determinant for teicoplanin resistance is *van*Z, which is generally found integrated as a *vanY-vanZ* locus next to the *vanA* cluster, conferring resistance to both vancomycin and teicoplanin^62^. Teicoplanin resistance is rarely present in strains that do not also exhibit vancomycin resistance. Among our clinical *E. faecium* VRE isolates, 40% were also resistant to teicoplanin. Of those showing teicoplanin resistance, 80% of the isolates harboured *vanY-vanZ* locus along with the *vanA* cluster. Presence of *vanZ* in *E. faecium* VREs exhibiting teicoplanin resistance is consistent with previous studies showing Tn1546-associated *vanZ*-mediated teicoplanin resistance in *E. faecium* BM4147^63^. However, further functional genetic investigations are required to determine the unexplained genetic basis of teicoplanin resistance in the remaining 20% of resistant isolates, as well as to determine the lack of teicoplanin resistance in *vanZ*-harbouring isolates.

Interestingly, the quaternary ammonium compound SMR family of drug efflux pump resistance genes *bcrB* and *bcrC* were only detected in isolates from the abattoir and retail meat. Quaternary ammonium compounds such as benzalkonium chloride are widely used as disinfectants in both food processing and medical environments^64^. Previously, these genes have been found in benzalkonium chloride-resistant *Listeria monocytogenes* H7550, a strain implicated in a 1998–1999 multistate outbreak originating from contaminated hot dogs^65^. In *L. monocytogenes*, these genes are present on a plasmid (pLM80) where they are associated with the IS1216 composite transposon. The presence of these genes on a transposon harbored by a plasmid raises the possibility of horizontal gene transfer via transposition or plasmid mobilization. As other genes associated with pLM80 could not be found in these isolates, the possibility of IS1216-based transposition cannot be ruled out. In *Enterococcus* spp., IS1216 transposons are also generally found in association with several other transposons, including Tn1546, Tn5385, Tn5482, and Tn5506, which may further promote horizontal gene transfer^66,67^. There is also a possibility that *L. monocytogenes* and *Enterococcus* spp. coexist and exchange genetic material within meat processing facilities, raising suspicion that these facility-adapted strains represent a greater food safety risk for meat products than those originating from cattle.

The majority of the virulence genes detected in this study were associated with capsular polysaccharide biosynthesis, biofilm formation, or adherence to surfaces. These genes are expected to be ubiquitous as they likely promote survival of *Enterococcus* spp. in both the GI tract and the environment. For example, capsular polysaccharide biosynthesis is associated with resistance to phagocytosis in humans and animals^68,69^, and increased fitness in aquatic environments due the capsule acting as a deterrent to both protozoa and bacteriophage^70,71^. Cytolysin production genes were frequently present in human clinical and urban wastewater *E. faecalis*, but were rarely present in isolates from other sources. Highly virulent strains of *E. faecalis* express cytolysin, a pore-forming exotoxin, which lyses both bacterial and eukaryotic cells in response to quorum signals. As the name reflects, cytolysin harbors dual bactericidal and cytolytic activities. It has been heavily implicated in the pathogenicity of *E. faecalis*. The association of cytolysin expression and increased toxicity of *Enterococcus* spp. infections has been studied in several animal models, as well as in clinical settings^72^. Cytolysin genes are generally located on pheromone-responsive *rep9*_pAD1/pTEF2/pCF10_ plasmids^73,74^. Our results suggested that pTEF2 was more frequently associated with wastewater and human clinical isolates. Cytolysin genes have also been reported to occur more frequently in clinical isolates (33%) compared to food isolates (6%)^75^. In our sequenced *E. faecalis*, only a few (catch basin, n = 2; surface water, n = 3; beef processing, n = 1) isolates associated with livestock or the environment harbored cytolysin genes, compared to their widespread abundance in wastewater and human clinical isolates. In the present study, we confirmed that the majority of livestock-associated *Enterococcus* spp. lacked virulence traits associated with clinical isolates. This result is encouraging and suggests that the risk of transmission of virulent *Enterococcus* spp. from cattle to humans is likely low.

Core genome phylogenomic comparisons also demonstrated that the majority of clinical *E. faecalis* and *E. faecium*, as well as urban wastewater isolates, differed from cattle and grouped as separate clades. Thus, urban wastewater isolates likely are reflective of human carriage. Such surveillance sites may be useful in future One Health studies, especially considering that wastewater is a rich source of fecal bacteria, and provides for monitoring of the fecal microbiota from large human populations without privacy concerns^76^. Similarly, MLST of genomes also supported the distinct nature of isolates from various sources with ST179 being the most prevalent sequence types for *E. faecalis*, and ST117 the most abundant for *E. faecium*. ST117 is a single locus variant of ST17, which constitutes one of the most common sequence types detected among clonal complex 17 (CC17) isolates worldwide^77^. In general, phylogenomics here revealed that most beef production and human-associated *E. faecalis* and *E. hirae* isolates were largely distinct, with limited sharing of, ARGs, virulence genes, and plasmids. Our findings are similar to a previous study^78^, where limited exchange of *E. faecium* strains and ARGs among livestock and humans was identified in the United Kingdom.

The One-Health approach undertaken in the present study improves our understanding of farm to fork AMR ecology associated with beef production. Niche-specificity makes enterococci a remarkable indicator bacteria with the potential adoption of similar approaches to other surveillance programs. Our findings suggest that specific *Enterococcus* spp. are tightly associated with specific host or environmental niches, and that dissemination of bovine-associated *Enterococcus* spp. beyond the feedlot is minimal. Substantial differences in the species profile between bovine fecal and human clinical isolates suggested that *Enterococcus* spp. from beef cattle may not play a significant role in *Enterococcus* spp. infections in humans. Furthermore, given that *E. hirae* was not abundant in environments downstream from beef cattle feedlots, waste flows appear to be prudently managed or *E. hirae* did not readily adapt to aquatic environments. However, judicious use of antimicrobial agents in animal production is essential to reduce selection for resistant *Enterococcus* spp. or other bacteria with zoonotic potential. Prudent use of antimicrobial agents in clinical settings is also needed to decrease the number of nosocomial *Enterococcus* spp. infections, considering that antimicrobial agents constitute a risk factor for these infections. A global policy on antimicrobial stewardship for both humans and animals is needed to curtail AMR transmission and spread.

## Materials and Methods

### Sampling

Four feedlots (A, B, C, D) with minimum operating capacities of ~15,000 cattle each, and their associated environments in Southern Alberta (Fig. 1A) were sampled for two consecutive years (March 2014 – April 2016). As previously described^79^, production conditions were typical for western Canadian commercial cattle feedlots, with animals housed in open-air, dirt-floor pens arranged side-by-side with central feed alleys. Fecal sampling was conducted according to a protocol approved by the Animal Care Committee, University of Calgary (Protocol ID: AC14–0029). From each feedlot, fecal samples were collected every two months from 20 different pens (100–300 cattle/pen), with the same 20 pens sampled throughout the study. Feces (~10 g) were sampled from 20 fecal pats and combined to generate a composite pen sample and transported in Cary-Blair enteric transport medium (BD Canada, Inc., Mississauga, ON). Samples were immediately refrigerated upon arrival in the lab and processed within 24 h after collection at the Agriculture and Agri-Food Canada (AAFC) Lethbridge Research and Development laboratory. Antimicrobials used in feedlots are summarized in Supplementary File 1, Table S6.

Wastewater was sampled from feedlot catch basins, which captured surface runoff from cattle pens. Wastewater samples were also obtained from a constructed wetland at feedlot C, which periodically received accumulated runoff from the catch basins. Surface water samples were obtained from a natural drainage/ephemeral creek adjacent to manured silage fields at feedlot C. Likewise, upstream and downstream surface water samples were obtained from an ephemeral creek that ran adjacent to feedlot A. Water samples (1L) were collected using a polyethylene bottle attached to a telescopic pole at four different locations per site, and combined to generate composite samples. Sampling locations remained consistent throughout the study. Samples were maintained on ice during transport and processed within 24 h after collection.

Composite core soil samples were collected from two agricultural fields adjacent to feedlot C once every year over 2 years of above described sampling period and included the following sample types: a field with no history of manure application (East field), from the same field but ~6 months after manure application, and from a field (West field) with a continuous history of manure application. Soil sampling was carried out using a soil coring kit (5 cm diameter) to a depth of 10 cm, and samples at 5–10 points along a 100 m transect were collected. A small number of samples (n = 5) were collected from stockpiled manure at the East field that was later to be applied to the field.

Samples were also collected from a beef processing plant. Sites sampled within the abattoir included carcass after hide removal, carcass after final wash, and conveyor belts. For sampling abattoir sites, 2 × 2 inch sterile gauze pads moistened with 0.1% peptone were used to swab the sampling area/carcass and then placed in a sterile stomacher bag for transportation. As part of beef processing, ground beef samples from abattoir and retail beef samples from regional retailers were also packed on ice and transported to the lab for culturing and isolation of *Enterococcus* spp.

To complement the cattle and environmental sampling and to represent the public element of the One Health continuum, two urban wastewater treatment plant sites in Alberta were sampled. These plants were located upstream and downstream of the major feedlot cattle production sites in the province. One liter each of composite influent (post-grit tank) and effluent (immediately prior to release) wastewater treatment samples were collected multiple times from the two municipalities over the same two year period.

In addition, human clinical *Enterococcus* spp. isolates were obtained through the Division of Medical Microbiology, Calgary Laboratory Services (CLS). CLS is a large regional consolidated laboratory that provides diagnostic services to Calgary and surrounding rural areas that encompass a population of ~1.5 million people. A total of 1,892 human *Enterococcus spp*. isolates recovered from clinical infections were collected/retrieved by CLS for this study from Jan. 2014 through to July 2016. All isolates were from individual patients and duplicates were excluded. All types of *Enterococcus spp*. recovered from humans were included. Clinically non-sterile site/source (NSS) isolates were collected prospectively from the urine and wound cultures while sterile site/source (SS) isolates (i.e., blood culture and sterile fluid sources) and vancomycin-resistant enterococci (VRE) were retrieved retrospectively from CLS’s extensive clinical biorepository of stored and well characterized isolates. Frozen isolates were processed in batches of 50 and each shipment to the research laboratory included 243 individual clinical isolates. The frozen isolates were sub-cultured to Tryptic Soy Agar with 5% Sheep Blood (Dalynn Biologicals, Dalynn, Calgary, Alberta), incubated at 37 °C in CO_2_ atmosphere for 18–20 h. All *Enterococcus* isolates were identified using a combination of methods that included phenotypic tests and Matrix-Assisted Laser Desorption Ionization-Time of Flight Mass Spectrometry (MALDI-TOF MS). After incubation, isolates were assigned anonymous study numbers and frozen in Tryptic Soy Broth with 15% Glycerol (Dalynn) before being shipped to the research laboratory using appropriate Transportation of Dangerous Goods (TDG) procedures. Clinical isolates were immediately stored at −80 °C at the research laboratory until sub-cultured for further testing.

### Bacterial isolation from cattle feces and environmental samples

Using the attached ‘spoon-lid’, fecal samples collected in Enteric Transport Vials with Cary-Blair medium were mixed to create a uniform slurry within the transport medium. A sterile cotton swab was completely immersed into the fecal slurry, and streaked in parallel onto Bile Esculin Azide (BEA) Agar without the addition of any antibiotic (designated here as antimicrobial-free or non-selective medium) and onto BEA supplemented with 8 µg/mL erythromycin (BEA + Ery; selective medium). The erythromycin concentration was based on the resistance breakpoint value established by the Clinical and Laboratory Standards Institute^80^. After 48 h incubation at 37 °C, presumptive *Enterococcus* spp., identified as esculin-hydrolyzing, white-tan-yellow colored, small-medium sized colonies, were selected and sub-cultured onto the same media to obtain pure cultures. For each sample, 3 isolates were collected per media type (BEA and BEA + Ery). Growth from subcultures was suspended in brain heart infusion (BHI) broth containing 15% glycerol and archived at −80 °C for subsequent characterization.

*Enterococcus* from feedlot catch basins, wetland and creek surface water, and urban wastewater (influent and effluent) were isolated by initially filtering various volumes of water samples (depending on the turbidity) through 0.45 µm nitrocellulose membranes that were placed onto modified mEI (BD Difco) agar according to US EPA Method 1600^81^, as well as mEI containing 8 μg/mL erythromycin. Plates were incubated at 37 °C for 48 h and presumptive enterococci colonies were sub-cultured onto BEA and BEA + Ery respectively, and archived as above.

*Enterococcus* were isolated from soil and manure by mixing 25 g of the sample with 100 mL of phosphate buffered saline (PBS), vortexing for 30 s, settling for 15 s, and filtration using sterile 3 mm Whatman filter paper. Ten-fold volumes (manure = 200 µL, 20 µL; soil = 500 µL, 20 µL) were plated onto *Enterococcus* specific/selective media BEA or mEI. Soil samples were enriched in parallel by incubating a 5 g sample in Enterolert (IDEXX Laboratories Canada Corp. Markham, ON, Canada) for 12 h, and streaking 5 µL onto mEI followed by incubation at 35 °C overnight.

*Enterococcus* isolates were recovered from beef slaughter and retail meat following an enrichment process as follows: For gauze pads 10 mL of Buffered Peptone Water (BPW; BD Difco) was added to each bag and stomached for 2 min. Ground beef (25 g) was weighed into a sterile filter bag, 225 mL of BPW was added and stomached for 2 min. One millilitre of liquid was drawn from each sample and added to 9 mL of double-strength Enterococcal enrichment Broth (EB; Dalyyn Biologicals Inc, Calgary AB, Canada) before incubating at 35 °C for 18–24 h. Positive colonies with brownish-blackish broth coloration were streaked on BEA and BEA + Ery plates using a sterile loop, and incubated at 37 °C for 48 h followed by subculture of up to 3 colonies from each media type, and archiving as glycerol stocks as described above.

### Enterococcus species identification

All presumptive *Enterococcus* spp. were identified via sequencing of the *groES-EL* intergenic spacer region as previously described^82^. Briefly, single colonies were suspended in TE buffer, heat-lysed at 95 °C for 5 min, and supernatant was used as the PCR template for species identification using Ent-ES-211–233-F (forward) and Ent-EL-74–95-R (reverse) primers. The PCR product was purified using QIAquick PCR purification kit, (Qiagen, Toronto, ON, Canada), and Sanger cycle-sequenced (Eurofins Genomics, Toronto, ON, Canada) using the above oligonucleotide.

The method for identifying *Enterococcus* spp. originating from bovine fecal samples was modified to include a multiplex PCR targeting the *groES-EL* intergenic region and *E. hirae*-specific muramidase gene (*mur-2*)^83^ to distinguish *E. hirae*. The multiplex PCR reaction mixture (25 μL) included 2 µL of template DNA from heat-lysed bacterial cells, 1 µM each of Ent-ES-211–233-F and Ent-EL-74–95-R primers, 0.25 µM each of mur2h_F1 (5′-TATGGATACACTCGAATATCTT-3′) and mur2h_R (5′-ATTATTCCATTCGATTAACTGC-3′) oligonucleotides and Qiagen HotStarTaq Plus Master Mix kit reagents as per manufacturer’s instructions. PCR condition were: 95 °C for 5 min, followed by 35 cycles of 94 °C for 30 sec, 49 °C for 30 sec, 72 °C for 30 sec, and a final extension at 72 °C for 10 min. Isolates with positive amplification for both *groES-EL* (~220 bp) and *mur-2* (483 bp) were identified as *E. hirae*. Isolates that were *groES-EL* positive and *mur-2* negative were Sanger sequenced using Ent-ES-211–233-F and Ent-EL-74–95-R. *Enterococcus* spp. were identified by BLAST search against a custom database of the *Enterococcus* spp. *groES-EL* intergenic region. Isolates negative for both *groES-EL* and *mur-2* were designated as non-*Enterococcus* and discarded.

### Antimicrobial susceptibility profiling

Detailed antimicrobial susceptibility testing (AST) was carried out on 25% of the study isolates (n = 2,110/8,430) and included 1,318 *E. hirae*, 375 *E. faecalis*, and 417 *E. faecium*. Considering that *E. hirae* predominated (n = 4,423) in feedlots (bovine feces and catch basin), about one-third (n = 1,264) of the isolates from this species across feedlots contributed to isolates tested for antimicrobial susceptibility. Other *E. hirae* tested included 46 isolates from surface water and 9 isolates from wastewater. AST was performed on most isolates collected from urban wastewater (n = 190; 91%). The remainder of the AST-profiled *E. faecalis* and *E. faecium* originated from surface water (n = 93). AST was also performed on all *E. faecalis* (n = 61) and *E. faecium* (n = 109) collected from feces and the catch basins. Only a small subsets of all of the *Enterococcus* isolates from human clinical cases were selected for AST in order to verify the antibiogram profiles previously obtained from CLS as part of the patient’s clinical care. These included a subset of 152 randomly selected NSS isolates including *E. faecalis* (n = 78) and *E. faecium* (n = 24) and all *Enterococcus spp*. for which a species-level identification was not obtained (n = 52). Almost all SS isolates including *E. faecalis* (n = 30) and *E. faecium* (n = 9), and the majority of VRE (n = 149) were AST profiled.

Disk susceptibility tests were conducted according to the Clinical and Laboratory Standards Institute (CLSI) documents M02-A12^84^ and M100-S24^80^. All *Enterococcus* isolates were tested against three major categories of antimicrobials based on their importance in human medicine^85,86^. Antimicrobials of critical importance included levofloxacin, linezolid, quinupristin/dalfopristin, teicoplanin and vancomycin; high importance drugs included erythromycin, ampicillin, gentamicin, streptomycin and medium importance drugs included nitrofurantoin, tetracycline, and tigecycline. Multidrug resistance was defined as resistance to two or more different classes of antimicrobials. *Staphylococcus aureus* ATCC 25923 and *E. faecalis* ATCC 29212 were used as reference quality controls. Zones of inhibition were read using the BioMic V3 imaging system (Giles Scientific, Inc., Santa Barbara, CA, USA) and categorized as sensitive or resistant based on CLSI interpretive criteria, except for tigecycline which used EUCAST interpretive criteria (The European Committee on Antimicrobial Susceptibility Testing, 2014).

### Whole-genome sequencing and data analysis

Across the continuum, a total of 708 *E. faecalis* and *E. faecium* isolates were selected for whole genome sequencing (WGS). Considering the importance of *E. faecium* and *E. faecalis* in human health, sequencing efforts were focused on only these two species and included 89 bovine feces, 44 catch basin wastewater, 39 beef processing, 68 surface water and 169 urban municipal wastewater isolates. The remaining 299 isolates represented the following: human clinical enterococci with 149 NSS isolates including 99 *E. faecalis* and *E. faecium* and 50 unidentified species; 36 SS *E. faecalis* and *E. faecium*; and 114 VRE (all *E. faecium*). *Enterococcus spp*. were streaked on to BEA plates from frozen glycerol stocks and incubated at 37 °C for 24 h to obtain typical esculin-hydrolyzing colonies that formed black or brown precipitates. A single colony was sub-cultured onto BHI agar (Dalynn Biologicals, Calgary, AB, Canada) and grown overnight at 37 °C. Bacterial growth was suspended in TE (10 mM Tris – 1 mM EDTA), pH 8.0 buffer at an OD_600_ of ~2 in order to obtain 2 × 10^9^ cells/mL of suspension. Cell suspension (1 mL) was transferred to a microcentrifuge tube and centrifuged for 2 min at 14,000 × g. Genomic DNA was extracted using a modified DNeasy Blood and Tissue Method (Qiagen, Montreal, QC, Canada). Briefly, bacterial cells were incubated for 1 h at 37 °C with shaking at 300 rpm in 280 µL of lysis buffer consisting of 20 mM Tris-HCl (pH 8.0), 2 mM sodium EDTA, Triton X-100 (1.2%), and 20 mg/mL lysozyme (Sigma Aldrich Canada, Toronto, ON, Canada). Subsequently, 35 µL of proteinase K and 5 µL of 100 mg/mL RNase A were added, and the mixture was incubated at room temperature for 20 min followed by the addition of 300 µL of absolute ethanol, and vortexing. The mixture was then added to the DNeasy Mini spin column and the rest of the steps were performed following manufacturer’s procedure. DNA quantity was estimated using a Nanodrop 2000 spectrophotometer and a Qubit Fluorometer with PicoGreen (Thermo Fisher Scientific, Mississauga, ON, Canada). Genomic library construction was performed using the Illumina Nextera XT DNA sample preparation kit (Illumina Inc., San Diego, CA, USA). Libraries were sequenced on an Illumina MiSeq platform using the MiSeq Reagent Kit V3 to generate 2 × 300 base paired-end reads.

Phylogenomic analysis of the whole genome sequence data was conducted via single nucleotide variant (SNV) phylogenomics (SNVPhyl v 1.0)^87^ of core genes using unassembled sequence read data. Briefly, paired-end reads originating from Illumina sequencing of samples were aligned to the reference genomes of *E. faecalis* (strain ATCC 47077/OG1RF; GenBank accession# CP002621.1) and *E. faecium* (strain DO; GenBank accession# CP003583.1) to generate read pileups (SMALT v.0.7.5; http://www.sanger.ac.uk/science/tools/smalt-0). Read pileups were subject to mapping quality filtering (minimum mean mapping quality score of 30), coverage cut offs (15X minimum depth of coverage), and a single nucleotide variant (SNV) abundance ratio of 0.75 to produce a multiple sequence alignment of SNV-containing sites. No SNV density filtering was performed on detected SNVs and the SNV-alignment was used to create a maximum-likelihood-based phylogeny using PhyML^88^. The resulting tree was visualized using FigTree v1.4.4 (http://tree.bio.ed.ac.uk/software/figtree/).

Sequencing reads were assembled *de novo* into contigs using the Shovill pipeline (https://github.com/tseemann/shovill). This pipeline included a quality trimming step with Trimmomatic 0.38 to remove common Illumina adapter sequences followed by *de novo* assembly with SPAdes version 3.13.0.^89^. Draft genome assemblies were annotated with Prokka^90^. ABRicate version 0.8.7 (https://github.com/tseemann/ABRICATE) was used to search contigs against the NCBI Bacterial Antimicrobial Resistance Reference Gene Database (NCBI BioProject ID: PRJNA313047) to identify ARGS, VFDB [pmid 15608208] for virulence factors, and the PlasmidFinder database version 2018-11-20^91^ to identify plasmids.

### Genome sequence data availability

The draft whole genome sequence assemblies of the 708 *Enterococcus* spp. have been deposited in GenBank under BioProject PRJNA604849.

### Ethics approval and consent to participate

Sampling procedures were reviewed and approved by the Lethbridge Research Centre Animal Care and Use Committee (AC# 14–0029), and were conducted according to the Canadian Council of Animal Care Guidelines. Feedlots were recruited for participation by Feedlot Health Management Services (FHMS), Okotoks, Alberta, Canada, and the owners of the participating feedlots provided explicit permission in accordance with provincial agriculture operation practices to work on the premises and to collect samples from feedlots that were enrolled in the study. Operators of the two municipal sewage treatment plants collected samples on our behalf and shipped samples to the Lethbridge Research and Development Center laboratory. No patient identifiers were recorded with isolated clinical enterococci and clinical isolates were assigned a new identification upon transfer from the clinical to the research laboratory to ensure complete anonymity. Only meta data with regard to time and site of sample collection were retained.

## Supplementary information

Supplementary information.

Supplementary information S1.

Supplementary information S2.

Supplementary information S3.
