# Author Correction: Surveillance of *Enterococcus spp*. reveals distinct species and antimicrobial resistance diversity across a One-Health continuum

**DOI:** 10.1038/s41598-020-69044-5

**Published:** 2020-08-04

**Authors:** Rahat Zaheer, Shaun R. Cook, Ruth Barbieri, Noriko Goji, Andrew Cameron, Aaron Petkau, Rodrigo Ortega Polo, Lisa Tymensen, Courtney Stamm, Jiming Song, Sherry Hannon, Tineke Jones, Deirdre Church, Calvin W. Booker, Kingsley Amoako, Gary Van Domselaar, Ron R. Read, Tim A. McAllister

**Affiliations:** 1 Lethbridge Research and Development Centre, Agriculture and Agri-Food Canada, 5403 1st Avenue South, Lethbridge, AB T1J 4P4 Canada; 2 Alberta Agriculture and Forestry, 100, 5401 1st Avenue South, Lethbridge, AB T1J 4V6 Canada; 3 Canadian Food Inspection Agency, National Center for Animal Disease, Lethbridge Laboratory, Township Rd 9-1, Lethbridge, AB T1J3Z4 Canada; 4 National Microbiology Laboratory, Public Health Agency of Canada, 1015 Arlington Street, Winnipeg, MB R3E 3R2 Canada; 5 Feedlot Health Management Services, Okotoks, AB Canada; 6 Lacombe Research and Development Centre, Agriculture and Agri-Food Canada, 6000C and E Trail, Lacombe, AB T4L 1W1 Canada; 7 Cumming School of Medicine, University of Calgary, 3280 Hospital Drive NW, Calgary, AB Canada; 8 Calgary Laboratory Services (CLS), Alberta Health Services, 3535 Research Rd NW, Calgary, AB T2L 2K8 Canada

Correction to:* Scientific Reports*10.1038/s41598-020-61002-5, published online 03 March 2020

This Article contains multiple typographical errors.

In the Results section under subheading ‘Sampling feedlot and downstream environment, a beef processing plant, retail meat and urban wastewater’.

“Urban wastewater contributed 31 samples in total including composite influent (post-grit tank; n = 22) and effluent (immediately prior to release; n = 21), which were collected over the same two year period as the fecal samples.”

should read:

“Urban wastewater contributed 43 samples in total including composite influent (post-grit tank; n = 22) and effluent (immediately prior to release; n = 21), which were collected over the same two year period as the fecal samples.”

In the Results section under subheading ‘Enterococcus recovery’,

“A total of 8,307 presumptive *Enterococcus* spp. isolates were recovered from all sites/sources tested including bovine feces (n = 4,499), feedlot catch basins (n = 510), surface water/natural water sources (n = 521), urban wastewater influent and effluent (n = 222), beef processing (abattoir and retail beef) (n = 774), and human clinical cases (n = 1,849) (Fig. 1B).”

should read:

“A total of 8,375 presumptive *Enterococcus* spp. isolates were recovered from all sites/sources tested including bovine feces (n = 4,499), feedlot catch basins (n = 510), surface water/natural water sources (n = 521), urban wastewater influent and effluent (n = 222), beef processing (abattoir and retail beef) (n = 774), and human clinical cases (n = 1,849) (Fig. 1B).”

“Isolate recovery rates from surface water, wastewater effluent and meat processing samples was lower for antibiotic-free as compared to non-selective media (Supplementary File 1, Table S2).”

should read:

“Isolate recovery rates from surface water, wastewater effluent and meat processing samples was lower for antibiotic-selective as compared to non-selective media (Supplementary File 1, Table S2).”

