# Supplementary material for: Surveillance of Enterococcus spp. reveals distinct species and antimicrobial resistance diversity across a One-Health continuum
Source: Sci Rep. 2020 Mar 3;10:3937. doi: 10.1038/s41598-020-61002-5 (PMC7054549; doi:10.1038/s41598-020-61002-5)
Supplement: Supplementary file 1 — Supplementary information. [file 41598_2020_61002_MOESM1_ESM.docx]

**Surveillance of *Enterococcus spp.* reveals their distinct species and antimicrobial resistance diversity across the One-Health continuum**

Rahat Zaheer^1^, Shaun R Cook^2^, Ruth Barbieri^1^, Noriko Goji^3^, Andrew Cameron^1^, Aaron Petkau^4^, Rodrigo Ortega Polo^1^, Lisa Tymensen^2^, Courtney Stamm^5^, Jiming Song^5^, Sherry Hannon^5^, Tineke Jones^6^, Deirdre Church^78^, Calvin W Booker^5^, Kingsley Amoako^3^, Gary Van Domselaar^4^, Ron R Read^7^, Tim A McAllister^1*^

^1^Lethbridge Research and Development Centre, Agriculture and Agri-Food Canada, 5403 1^st^ Avenue South, Lethbridge, AB, T1J 4P4, Canada

^2^Alberta Agriculture and Forestry, 100, 5401 1^st^ Avenue South, Lethbridge, AB, T1J 4V6, Canada

^3^Canadian Food Inspection Agency, National Center for Animal Disease, Lethbridge Laboratory, Township Rd 9-1, Lethbridge, AB, T1J3Z4, Canada

^4^National Microbiology Laboratory, Public Health Agency of Canada, 1015 Arlington Street, Winnipeg, MB, R3E 3R2, Canada

^5^Feedlot Health Management Services, Okotoks, AB, Canada

^6^Lacombe Research and Development Centre, Agriculture and Agri-Food Canada, 6000 C and E Trail

Lacombe, AB, T4L 1W1, Canada

^7^Cumming School of Medicine, University of Calgary, 3280 Hospital Drive NW, Calgary, Alberta

^8^Calgary Laboratory Services (CLS), Alberta Health Services, 3535 Research Rd NW, Calgary, AB T2L 2K8 Canada.

* Correspondence to: [tim.mcallister@canada.ca](mailto:tim.mcallister@canada.ca)

**Supplementary Table S1:** Sampling details.

| **Location/Site** | | **Number of samples collected over 2 years** |
| --- | --- | --- |
| **Beef Cattle Feedlots (n=4)** | | |
|  | Composite Pen Fecal | 866 |
|  | Catch Basins | 72 |
|  | Manure stockpile | 5 |
| **Environment** | | |
|  | Surface water (constructed wetland, creek, drain, clean storage) | 57 |
|  | Soil | 25 |
| **Urban wastewater** | | |
|  | Influent | 22 |
|  | Effluent | 21 |
| **Beef Processing** | | |
|  | Carcass gauze swab after Hide Removal (abattoir) | 150 |
|  | Carcass gauze swab after Final Wash (abattoir) | 150 |
|  | Gauze swab Conveyor Belts (abattoir) | 150 |
|  | Ground Beef (abattoir) | 150 |
|  | Retail Meat | 60 |
|  | **Total number of samples** | **1728** |

**Supplementary Table S2**: Percentage of samples positive for enterococci per sampling site.

|  | **Enterococci** | |
| --- | --- | --- |
|  | ***antibiotic-free media*** | ***Selective media (with erythromycin)*** |
| **Feedlot – Pen Composite** | | |
| A | 99.56% | 98.25% |
| B | 98.66% | 81.25% |
| C | 98.60% | 92.52% |
| D | 99.50% | 91.50% |
| **Feedlot – Catch Basin** | | |
| A | 87.50% | 75.00% |
| B | 100.00% | 83.33% |
| C | 70.59% | 61.76% |
| D | 58.33% | 54.17% |
| **Surface Water** | | |
| C – Constructed Wetland | 100.00% | 68.42% |
| C – Clean storage | 100.00% | 71.43% |
| C – Drain | 100.00% | 12.50% |
| A – creek upstream feedlot | 81.82% | 27.27% |
| A – creek downstream feedlot | 100.00% | 45.45% |
| **Soil** | | |
| C – East Field | 0.00% | 0.00% |
| C – West Field | 0.00% | 0.00% |
| C – Manure stockpile | 100.00% | 100.00% |
| **Urban wastewater** | | |
| Medicine Hat – Influent | 100.00% | 100.00% |
| Medicine Hat – Effluent | 72.73% | 9.09% |
| Calgary – Influent | 100.00% | 100.00% |
| Calgary – Effluent | 90.00% | 20.00% |
| **Beef Processing** | | |
| Hide Removal | 59.33% | 32.67% |
| Final Wash | 15.33% | 8.00% |
| Conveyor Belts | 12.67% | 0.67% |
| Ground Beef | 82.67% | 35.33% |
| Retail Meat | 78.33% | 36.67% |

**Supplementary Table S3:** Proportion of *Enterococcus* species isolated from various sample types

[number(%)]

|  | **Fecal** | **Manure stockpile** | **Catch Basin** | **Surface Water** | **Abattoir - retail meat** | **Urban wastewater** | **Clinical** |
| --- | --- | --- | --- | --- | --- | --- | --- |
| ***E. avium*** | 4(0.09) | 0(0) | 0(0) | 0(0) | 1(0.13) | 0(0) | 0(0) |
| ***E. casseliflavus*** | 5(0.11) | 10(29.41) | 85(16.47) | 119(22.67) | 5(0.65) | 1(0.43) | 3(0.16) |
| ***E. columbae*** | 2(0.04) | 0(0) | 0(0) | 0(0) | 0(0) | 0(0) | 0(0) |
| ***E. durans*** | 91(2.02) | 0(0) | 35(6.78) | 89(16.95) | 14(1.81) | 9(3.86) | 0(0) |
| ***E. faecalis*** | 35(0.78) | 0(0) | 26(5.04) | 42(8.00) | 572(73.90) | 121(51.93) | 1541(83.34) |
| ***E. faecium*** | 73(1.62) | 0(0) | 45(8.72) | 52(9.90) | 34(4.39) | 89(38.20) | 249(13.47) |
| ***E. gallinarum*** | 1(0.02) | 0(0) | 18(3.49) | 4(0.76) | 7(0.90) | 4(1.72) | 1(0.05) |
| ***E. hirae*** | 4117(91.51) | 24(70.59) | 306(59.30) | 145(27.62) | 112(14.47) | 9(3.86) | 3(0.16) |
| ***E. malodoratus*** | 47(1.04) | 0(0) | 0(0) | 0(0) | 9(1.16) | 0(0) | 0(0) |
| ***E. mundtii*** | 5(0.11) | 0(0) | 1(0.19) | 74(14.10) | 3(0.39) | 0(0) | 0(0) |
| ***E. raffinosus*** | 0(0) | 0(0) | 0(0) | 0(0) | 17(2.20) | 0(0) | 0(0) |
| ***E. villorum*** | 119(2.65) | 0(0) | 0(0) | 0(0) | 0(0) | 0(0) | 0(0) |
| ***Enterococcus spp.*** | 0(0) | 0(0) | 0(0) | 0(0) | 0(0) | 0(0) | *52(2.81) |
| **TOTAL isolates:** | **4499** | **34** | **516** | **525** | **774** | **233** | **1849** |

*Fifty of these isolates were later characterized as *E. faecalis* (42), *E. faecium* (5), *E. casseliflavus* (1), *E. hirae* (1), and *E. raffinosis* (1).

**Supplementary Table S4:** Antimicrobial resistance genes identified in *E. faecalis*, and *E. faecium* genomes

| **Resistance type** | **No. of genes** | **Gene name(s)** |
| --- | --- | --- |
| aminoglycoside | 13 | ***aac(6')-Ie/aph(2'')-Ia****,* ***aac(6')****, aadE, aac(6')-Ii, aac(6')-Iid, aadD1,* ***ant(6)-Ia****, ant(9)-Ia, ant(9)-Ib, aph(2'')-IIIa,* ***aph(3')-IIIa****, str,* ***spw*** |
| quaternary ammonium compound small multidrug resistance (SMR) family of drug efflux pumps | 2 | *bcrB, bcrC* |
| linezolid | 1 | ***cfr*** |
| phenicol | 4 | ***cat-TC****,* ***catA****, catP,* ***fexA*** |
| oxazolidinone phenicol transferable resistance | 1 | ***optrA*** |
| macrolide | 4 | ***erm(A)****,* ***erm(B)****, erm(T),* ***msr(C)*** |
| lincosamide & streptogramin A | 5 | *lnu(A),* ***lnu(B)****,* ***lnu(G)****,* ***lsa(A)****,* ***lsa(E)*** |
| lincosamides, streptogramins A, and pleuromutilins | 1 | ***eat(A)*** |
| streptothricin | 1 | ***sat4*** |
| tetracycline | 5 | ***tet(L)****,* ***tet(M)****, tet(O),* ***tet(S)****, tet(W)* |
| trimethoprim | 4 | *dfrC,* ***dfrE****,* ***dfrF****,* ***dfrG*** |
| vancomycin gene cluster | 15 | *vanA, vanB,* ***vanC****, vanH-A, vanH-B,* ***vanR****,* ***vanS****,* ***vanT/Tc****, vanW, vanX-A, vanX-B,* ***vanXY-C****, vanY-A, vanY-B, vanZ-A* |

Genes in **bold** were detected in isolates from both species.

**Supplementary Table S5:** Virulence factors and their associated genes identified in *E. faecalis*, and *E. faecium* genomes

| **Virulence factors** | **No. of genes** | **Gene name(s)** |
| --- | --- | --- |
| adhesins | 2 | ***EF0149*, *sgrA*** |
| aggregation substances | 3 | ***EF0485****, asa1,* ***prgB/asc10*** |
| hyaluronidases | 2 | ***hylA*/EF0818***,* ***hylB*/EF3023** |
| biofilm-associated proteins | 8 | ***ace***, ***bopD***, ***gelE***, ***ebpA****,* ***ebpB****,* ***ebpC***, *esp*, ***srtC*** |
| lipopolysaccharide biosynthesis proteins | 2 | *bplB*, *bplC* |
| bile salt hydrolase | 1 | ***bsh*** |
| caseinolytic protease | 1 | ***clpP*** |
| capsular polysaccharide biosynthesis | 11 | ***cpsA***, ***cpsB***, ***cpsC***, *cpsD*, *cpsE*, ***cpsF***, *cpsG*, *cpsH*, ***cpsI***, ***cpsJ***, ***cpsK*** |
| cytolysin production | 8 | ***cylA***, *cylB*, *cylI*, *cylL*, *cylM*, *cylR1*, *cylR2*, *cylS* |
| collagen binding protein | 3 | ***ecbA****, acm, scm* |
| endocarditis specific antigen | 1 | ***efaA*** |
| virulence regulator/signal transduction system | 3 | *fsrA*, *fsrB*, *fsrC* |
| fibrinogen adhesins | 3 | ***fss1***, ***fss2***, ***fss3*** |
| fimbrial biogenesis | 2 | *pilT*, *pilU* |
| metal binding lipoprotein | 1 | ***psaA*** |
| serine protease | 1 | ***sprE*** |

Genes in **bold** were detected in isolates from both species.

**Supplementary Table S6:** Description of antimicrobials used in the feedlots

| **Antimicrobial family** | **Antimicrobial** | **Route** |
| --- | --- | --- |
| Tetracycline | Chlortetracycline | In Feed |
|  | Oxytetracycline | Parenteral |
| Ionophore | Monensin | In Feed |
|  | Lasalocid | In Feed |
| Macrolide | Tylosin | In Feed/parenteral |
|  | Tulathromycin | Parenteral |
|  | Tilmicosin | Parenteral |
| Phenicol | Florfenicol | Parenteral |
| Cephalosporin | Ceftiofur | Parenteral |
| Fluoroquinolone | Enrofloxacin | Parenteral |
| Potentiated sulfonamide | Sulfadoxine | Parenteral |
| Sulfonamide combination | Sulfanilamide, sulfathiozole, sulfamethazine | Oral Administration |

The in-feed antimicrobials (ionophores, chlortetracycline or tylosin) were administered to all cattle in the conventional feedlot throughout the feeding period, while the therapeutic parenteral drugs were administered to only clinically ill cattle as required. Tylosin was used in-feed at 11 mg/kg dry matter (DM) basis. In feed Chlortetracycline was used at 1g/100 lb = 10 mg/lb=20mg/kg. Sulfonamides had limited use and aminoglycosides were rarely used.


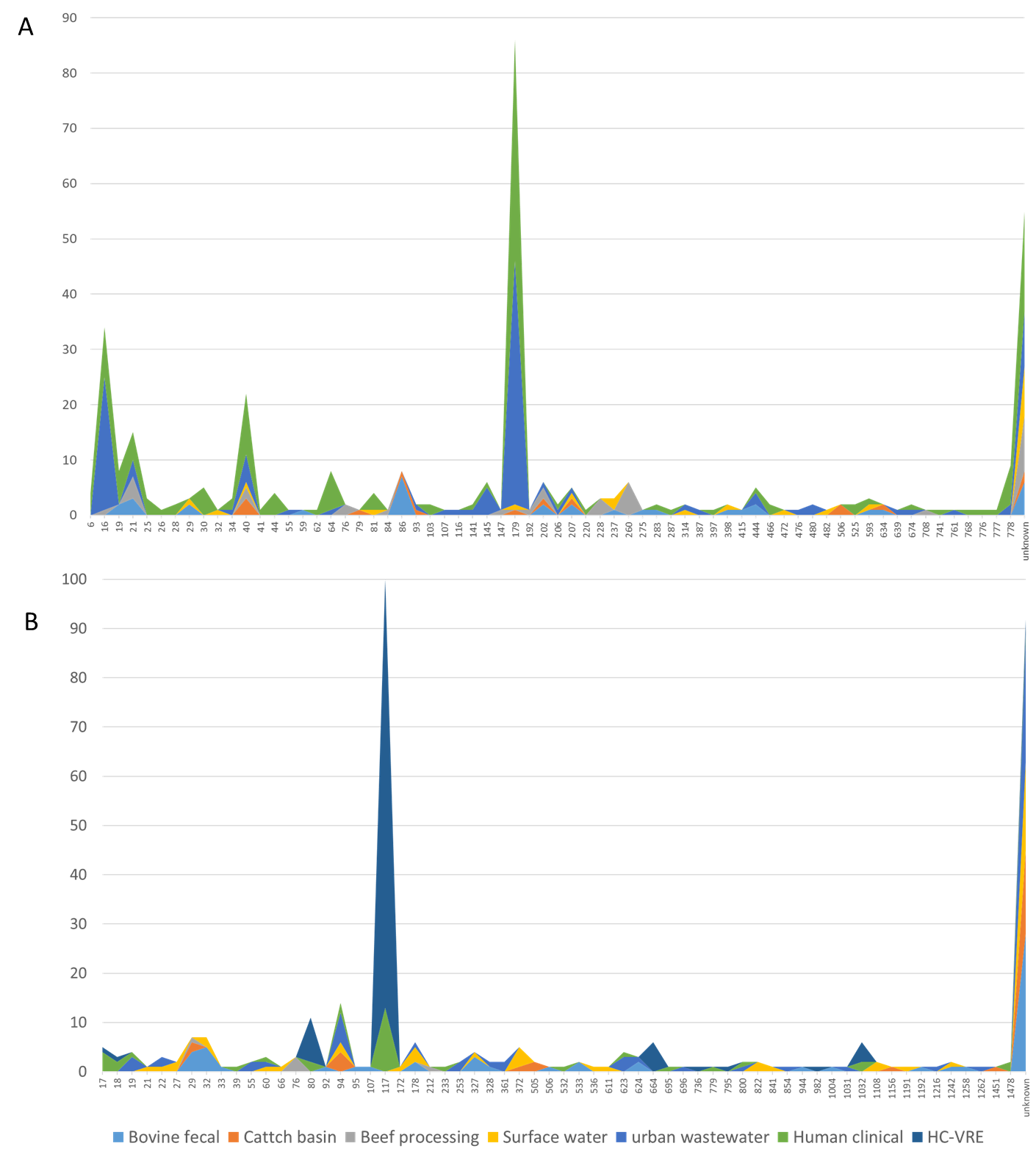


**Figure S1:** MLST profiles of *E. faecalis* (A) and *E. faecalis* (B) across the continuum


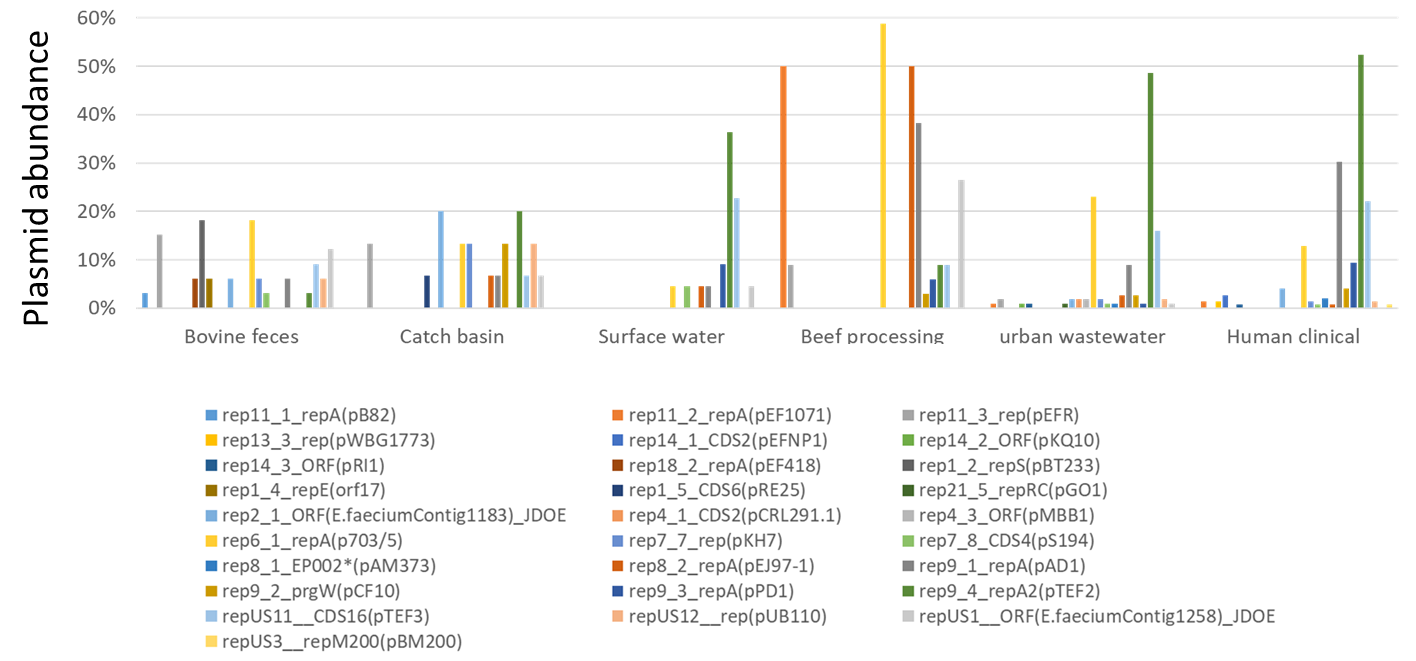


**Figure S2:** Abundance of various plasmids in *E. faecalis* across the continuum


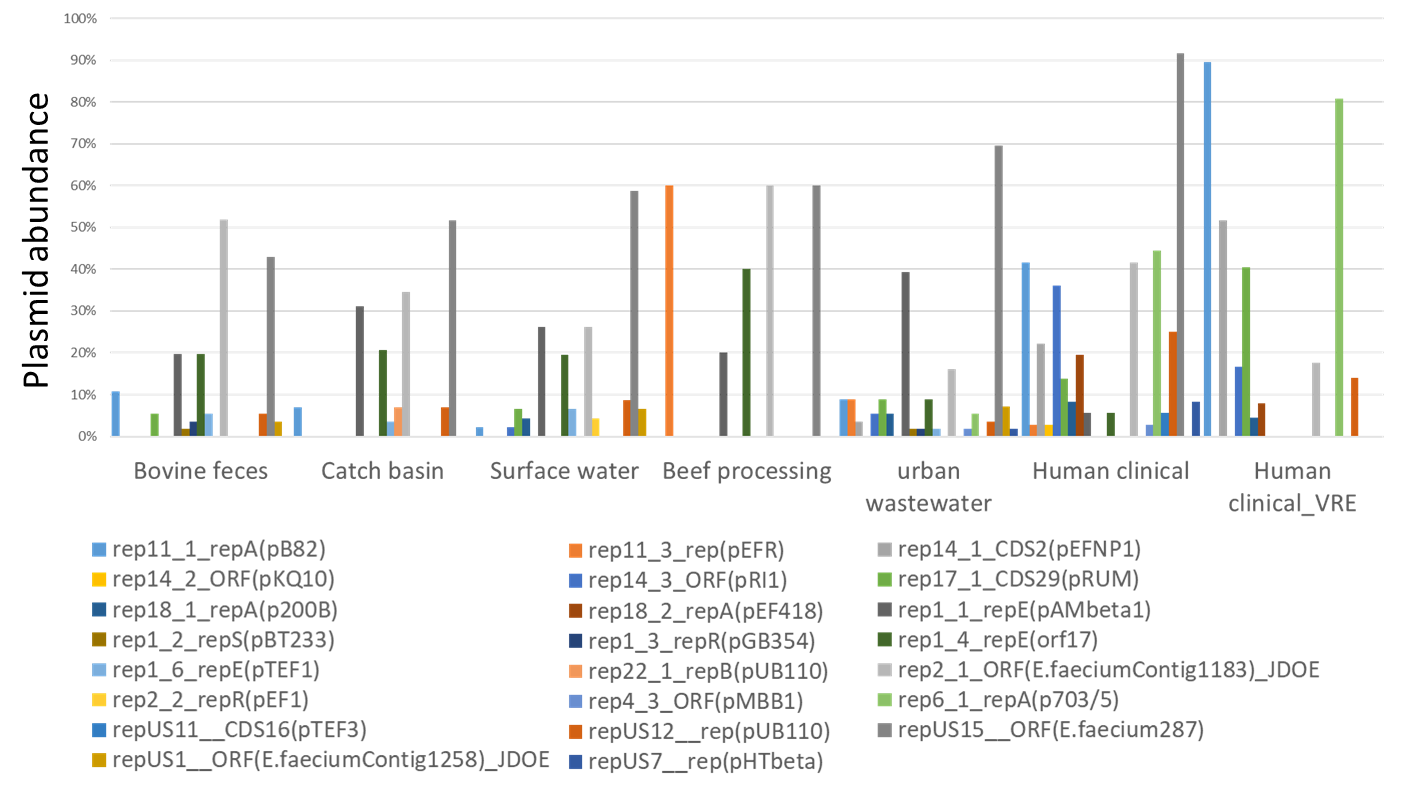


**Figure S3:** Abundance of various plasmids in *E. faecium* across the continuum
